# Supplementary material for: Relaxed constraint and functional divergence of the progesterone receptor (PGR) in the human stem-lineage
Source: PLoS Genet. 2020 Apr 17;16(4):e1008666. doi: 10.1371/journal.pgen.1008666 (PMC7190170; doi:10.1371/journal.pgen.1008666)
Supplement: Attachment — Submitted filename: Response to Reviewers.docx [file pgen.1008666.s003.docx]

The reviews were quite positive but have identified some areas that need clarification and further discussion. In addition to responding to the reviewer comments it would be useful to better place your results into the context of those from Chen 2008. Chen 2008 identified the human and chimpanzee clade together as providing the strongest evidence for positive selection. It would thus make sense to determine whether you get the same results (no positive selection, just relaxed selection) if you consider the human/chimp clade as the focal point compared to just the human-stem lineage as you have already done.

**Our revised manuscript now includes a section entitled “No evidence for positive selection in Hominini,” in which we use lineage specific methods (one and two ratio models, aBSREL, BUSTED, and RELAX) to characterize the strength and direction of selection acting on PGR in the human/chimp clade (Hominini). Similar to our other results (and those of Chen et al. 2008), we find that there is evidence for a rate acceleration in Hominini, but that there is no evidence of positive selection. In contrast, the RELAX test was significant, indicating that the rate acceleration is likely a result of relaxed selection intensity, rather than positive selection. We have updated Figure 1 to highlight the Hominini clade, and the supplementary tables to include the new aBSREL, BUSTED, and RELAX results.**

Comments to the Authors: (**Author responses in BOLD**)

Reviewer #1: Marinic and Lynch analyzed the molecular evolution of the human PGR gene by examining 119 Eutherian PGR sequences, including species and sub-species from each primate lineage, using multiple algorithms (shown in Table 1-4). Their findings suggest that PGR evolved under relatively strong purifying selection and that the human stem-lineage in particular shows evidence of rapid evolution. Their study resolves this issue which was uncertain based on previous analyses. Marinic and Lynch also examined the transcriptional activity of the ancestral human and human/chimp PR-A and PR-B isoforms using the human prolactin promotor driving a luciferase reporter in the context of mouse embryo fibroblast cells. Their results indicate that the ancestral human PR-B evolved to have stronger trans-activation whereas the transrepression of PR-A was less in the human PR-A than in the human/chimp PR-A. The authors conclude that the transcriptional activities of PR-A and PR-B may have diverged in the human lineage compared with non-human animals, although their data is limited to transcriptional activity based on the prolactin promoter in mouse cells. This is a well-written manuscript that brings important insight to understanding the distinctive actions of progesterone in human reproductive biology.

Critique:
1. Conclusions are skewed toward the isoform shifting hypothesis to explain how progesterone actions are withdrawn to trigger human parturition. This is based on PR activity at the prolactin promoter. However, PRs regulate the expression of multiple genes in reproductive cells that do not have a canonical PRE in their promotors. In this context the transrepressive activity of PR-A may not apply. This should be acknowledge in the discussion. Also the implication of the study to other distinctive actions of progesterone in human physiology (e.g., decidualization) could be discussed.

**These are excellent points. We now note in the Discussion that:**

**1) “We note, however, that PRs regulate the expression of genes without canonical progesterone response elements in their promoters, which may restrict the ability of PR-A to trans-repress PR-B.”**

**2) “Finally, while we have focused much of our discussion on how functional divergence of PR-A and PR-A relates to the isoform switching hypothesis, the progesterone receptor underlies multiple biological functions (such as decidualization), suggesting functional divergence may also affect those processes.”**

2. Figure 1: What dose 0.3 on the x-axis mean?

**The 0.03 on the x-axis is a scale bar for the branch lengths of the tree that indicates the number of substitutions per site, as a measure of sequence divergence (long branches are more diverged than shorter branches). We apologize for not including the “Subs per site” above the scale bar. We have updated the figure to include “Subs per site” and a description of this metric in the Figure 1 legend.**

3. Figure 2: Please define the Y-axis; what is bits?

**Bits are a measure of randomness; random sequences have a low bit score while non-random (conserved) sequences have a high bit score (max 4.2 for amino acid sequences). We have included this information in the Figure 2 legend.**

Reviewer #2: The authors of the manuscript “Relaxed constraint and functional divergence of the progesterone receptor (PGR) in the human stem-lineage” report divergent functions of the PR isoforms within the Catarrhine lineage that coincides with the detection of relaxed selection intensity. This report is significant given the role of progesterone in human reproduction and disease. Furthermore, our current understanding of the effects of the levels of progesterone and its receptors is limited. Within the introduction the authors elaborate on the significance and context of PR. Overall the authors present a compelling argument for the importance of understanding the evolution of PR. There are some specific issues within the introduction, however, that should be addressed.

1. The authors lay out the features of PR that are critical to understanding their results—such as the trans-activation and trans-repression. While the manuscript focuses on the evolution and function of the PR gene, there is some evidence that regulation of PR is also important and the authors give examples in the conclusions (PR-A/PR-B abundance section). Could evolution of PR-mediated regulation be regulatory? Do these experiments address this possibility?

**It is possible that changes in *PGR* transcript abundance compensate for changes in PR protein function. Our results do not address this possibility (indeed it would be difficult to address without gene expression information from other apes). We have added this to our Conclusions paragraph.**

2. The logic of the paragraph beginning “While female reproductive” is difficult to follow. The first section states that well conserved traits can have underlying genes that evolve rapidly. The second section states that lineages with divergent traits therefore should also have rapidly evolving genes. Are all reproductive traits likely to have rapidly evolving underlying genes? It is unclear how these examples “suggest that PR may have evolved rapidly”. Furthermore, it is unclear how the citations “Arbeitman et al. 2002, Meiklejohn et al. 2003 and Parisi et al. 2003” on Drosophila expression and gene evolution are associated with Eutherian and Therian reproductive gene evolution.

**I’m not sure statements about “all” things in some category are valid. Given the number of genes that are involved in reproduction, some genes will be conserved across species. That said, phenotypic traits that are divergent surely must have diverged in some aspects of the genes and gene regulatory networks that underlie them.**

**We used these citations as references to examples of reproductive genes evolving rapidly. We have reworded that sentence to read: “the genes that underlie reproductive traits in a wide range of taxa can evolve rapidly, often under the influence of positive selection” and modified choice of references to illustrate this better.**

3. The authors suggest that Catarrhine primates have “a divergent (and unknown) parturition signal” and yet in the discussion discuss at length the evidence for PR as a mechanism of parturition. The exact and entire mechanism is unknown but there is a large body of research into parturition.

**Indeed there is! Unfortunately, there is little evidence that progesterone or progesterone receptor levels directly influence human parturition. While a complete review of the role PR plays in human parturition is outside the scope of this manuscript, it is interesting that supplemental progesterone administration to women at elevated risk of preterm birth either has no effect at delaying parturition or does so only weakly, suggesting that progesterone levels near the end of human pregnancy may not influence birth timing. As I’m sure the reviewer knows, there is a clear role for inflammatory signals in the initiation of both term and preterm birth. Therefore, we have added this clarifying statement and included a reference to a review for interested readers.**

4. The authors thoroughly and insightfully cover the evidence for selection on PR along the human lineage. However, they do not discuss recent selection among human populations (Li et al 2018; “Natural Selection Has Differentiated the Progesterone Receptor among Human Populations”). How does evidence of both positive and balancing selection in recent human history support or contradict the findings in this manuscript?

**We thank the reviewer for pointing out this reference, of which we were unaware. We have included a new paragraph in the Discussion under the subsection entitled “Positive selection, relaxation, or both?” in which we discuss the results of Li et al. in light of our findings. Briefly, we think the results are complementary and suggest that, while the intensity of purifying selection is relaxed in primates, there is some evidence that selection is acting to reduce the frequency of early preterm associated variants in modern human populations.**

The authors set about to show that 1) PGR evolved rapidly in the human lineage; 2) Rate acceleration is consistent with relaxed purifying selection and not positive selection; and 3) Relaxed selection resulted in altered PR function. The methods used to test these hypotheses are robust and appropriately applied.

1. The authors use HyPhy to test for rapid evolution along the human lineage. The sentence starting “The two w model was significantly better…” is confusing. First, w2 is not defined (or detailed in the supplements) but I assumed it is associated with the stem-lineage (Figure 1 asterisk).

**You are correct, we have re-written the description of the two-ω model to make this clearer. It now reads: “We first compared two likelihood models using HyPhy (Kosakovsky Pond, Frost and Muse, 2005): a one-ratio model in which all lineages have a single *dN/dS* ratio (ω), and a two-ratio model in which the *dN/dS* ratio in the human stem-lineage (ω_1_) was estimated separately from the *dN/dS* ratio in all other lineages (ω_2_).”**

Second, it is not clearly laid out that this is the evidence for rapid evolution along the stem lineage. The authors may also want to define “rapid evolution” as an increase in the rate of nucleotide substitution compared to other lineages (as it is defined in human accelerated regions in Pollard 2006) to avoid confusion between absolute substitution rate and rate of evolution.

**This is an excellent idea, we have rewritten the last sentence of intro paragraph 2 to explicitly define what we mean by rapid evolution: “These data suggest that PR may have evolved rapidly in humans and Catarrhines (here we define “rapid evolution” as a lineage-specific increase in the relative rate of nucleotide substitution compared to other lineages *sensu* Pollard *et al*., 2006).”**

2. The authors present multiple lines of evidence to suggest that positive selection is not responsible for the rapid evolution of PGR. They account for multiple models of positive selection including episodic and weak selection. These results are reported in Tables 1 – 4. It would be useful to distinguish sites where there is significant evidence that w < 1 versus sites where there is no evidence that w doesn’t = 1. This would be helpful since each of the methods has different abbreviations and methods for assessing significance.

**This is a useful suggestion, we now include a panel in Figure 2 that summarizes the site-specific selection tests (FEL, SLAC, FUBAR, MEME).**

The authors also provide support the conclusion that relaxed selection is responsible for the elevated rate of evolution in PGR. This is represented in Figure 1. From the methods section and description, it is unclear if the phylogeny in Figure 1 is a species phylogeny (as suggested by the title “Phylogeny of species”) or a gene tree (only information from the PGR gene).

**We now specify in the first paragraph of the results section that the phylogeny shown in Figure 1 is the species phylogeny, not the PGR gene tree.**

3. The authors also present both computational and experimental evidence that human specific changes in PGR are functionally important. The computational results using CADD suggest that the human-specific substitutions are deleterious. The likelihood of functional consequences from these changes is further supported by luciferase assays. Figure 3D would be strengthened if the p-values were reported in the figure or legend.

**We now include p-values for comparisons shown in Figure 3D within the figure legend.**

Overall the methods presented in the manuscript strongly support the conclusions of the authors. The only concern is the presentation of the evidence for “rapid evolution.” The authors demonstrate how the use of multiple measures/tools can help more precisely differentiate between modes of selection that are often difficult to distinguish.

**We hope that explicitly defining “rapid evolution” as suggested by the reviewer allays this concern.**

The supplemental excel data covers almost all of the experiments conducted except the TestBranchDNDS data and the raw luciferase data. The authors should also consider providing the PGR sequences they identified and/or assembled.

**We now include the luciferase data in the supplementary excel tables, and the PGR alignment/tree as a supplementary text file (PGR.fasta). The output of the TestBranchDNDS analyses is given in the text (no other information is included in the output).**

In the discussion the authors summarize their results and place them in the context of PRs role in parturition. Most importantly the authors discuss how changes in repressor strength in PR-A may be involved in the differences between human parturition and that of common model organisms such as rodents and even close relatives such as chimps. The manuscript is well written but should be clarified in a few locations noted above. It is striking how little we know about human parturition and pregnancy—this manuscript meaningfully contributes to improving our understanding of these important topics.
